# Use of a Risk Assessment Model for Venous Thromboembolism Is Associated with Decreased Prophylaxis

**DOI:** 10.1007/s11606-025-09592-6

**Published:** 2025-05-08

**Authors:** Tarini Gunaratne, Rebecca Schulte, Stephanie Moss, Oleg Lisheba, Michael B. Rothberg

**Affiliations:** 1 Department of Internal Medicine, Ochsner Medical Center, New Orleans, LA USA; 2 Quantitative Health Sciences, Cleveland Clinic, Cleveland, OH USA; 3 Department of Hospital Medicine, Cleveland Clinic, Cleveland, OH USA; 4 Enterprise Analytics eResearch Department, Cleveland Clinic, Cleveland, OH USA; 5 Center for Value-Based Care Research, Cleveland Clinic, 9500 Euclid Avenue, Cleveland, OH G1044195 USA

**Keywords:** venous thromboembolism prophylaxis, clinical decision support, hospital medicine

## Abstract

**Background:**

Venous thromboembolism (VTE) prophylaxis is often overprescribed to patients at low risk for VTE. Whether risk assessment models (RAMs) reduce prescribing to low-risk patients is unknown. We incorporated a validated RAM into admission order sets to help physicians determine risk of VTE.

**Objective:**

To quantify RAM use, determine its association with prophylaxis, and identify patient factors associated with concordance between calculated VTE risk and prophylaxis use. We hypothesized that use of the RAM would be associated with less prophylaxis.

**Design:**

Cross-sectional study. We excluded surgical, COVID, and intensive care unit patients, and patients with contraindication to prophylaxis or already on anticoagulation.

**Participants:**

Medical inpatients aged ≥18 years admitted to 10 US hospitals from December 2020 to March 2023.

**Interventions:**

Physician RAM use.

**Main Measures:**

Physician prophylaxis prescription and patient characteristics.

**Key Results:**

Among 131,441 patient encounters, RAM use varied across hospitals from 54 to 99%. Overall, physician ordering was concordant with the RAM’s recommendation for 68% of patients. Prophylaxis prescription was less common when the RAM was used than when it was not (44% vs. 73%, *p* < 0.001). When calculated risk was high (i.e., >0.75%), 96% of patients had prophylaxis prescribed versus 37% when risk was low. Across hospitals, prophylaxis prescription rates varied more for low-risk (21 to 77%) than for high-risk patients (87 to 98%). Among low-risk patients, prophylaxis was associated with male sex, older age, reduced mobility, and history of DVT, stroke, heart or respiratory failure, or active cancer.

**Conclusions:**

Use of the RAM was associated with reduced prophylaxis prescribing, but many low-risk patients still received prophylaxis, especially if they had a risk factor for VTE. Physicians appear to agree with high-risk assessments but are less comfortable not prescribing prophylaxis to patients at low risk.

**Supplementary Information:**

The online version contains supplementary material available at 10.1007/s11606-025-09592-6.

## INTRODUCTION

Acutely ill patients are at increased risk of developing venous thromboembolism (VTE), which can be prevented through the use of chemoprophylaxis.^1^ However, prophylaxis is not without harms, and both the American Society of Hematology (ASH)^2^ and the American College of Chest Physicians^3^ limit their recommendation to prescribe low molecular weight heparin, unfractionated heparin, or fondaparinux to acutely ill medical patients who are at high risk of hospital-acquired VTE. Reasons to avoid prophylaxis in low-risk patients include patient discomfort, heparin-induced thrombocytopenia (HIT), bleeding, and unnecessary use of resources, including nurse and pharmacist time and medication costs.^4,5^ Nevertheless, after decades of quality improvement efforts aimed at increasing prophylaxis for high-risk patients, overtreatment of low-risk patients is now widespread, with estimates of prophylaxis among low-risk patients ranging from 74^6^ to 87%.^7^

To help physicians perform risk stratification, a number of prediction models have been developed.^8^ These include the Padua^9^ and IMPROVE^10^ risk assessment models (RAM), both of which have been externally validated^11^ and are recommended by ASH.^2^ However, there are few examples of implementation of these models in clinical care. Moreover, numerous studies have demonstrated that when models are incorporated into the EHR to identify high-risk patients, physicians usually ignore the ensuing alerts. Using clinical decision support to avoid prophylaxis for low-risk patients has been less studied. Given the emphasis placed on VTE prophylaxis, it is not clear how receptive physicians would be to recommendations not to give it.

In 2017, we developed a RAM using local EHR data from more than 160,000 patients. It includes 12 risk factors and employs logistic regression to predict a patient’s probability of VTE, which is used to determine whether the patient is low risk or high risk. The RAM was externally validated at 52 Michigan hospitals and outperformed the IMPROVE and Padua models.^12^ The RAM was then tested in a cluster-randomized trial.^13^ In that trial, physicians mostly chose not to use the RAM, but when they did, they prescribed more targeted prophylaxis. Following conclusion of the trial, the RAM was incorporated into the admission order set for all Cleveland Clinic hospitals including a “hard stop,” so that physicians had to acknowledge the calculated risk before proceeding to enter orders. Physicians are not obligated to follow the RAM’s recommendations.

Previous studies of clinical decision support for VTE prophylaxis found that physicians override or ignore recommendations most of the time.^14–16^ Our RAM differs from past approaches because physicians acknowledge the calculated risk before proceeding to order prophylaxis. When decision support is presented in this way, it is not known how often physicians will use it or whether they will agree with its assessment. Our study had four objectives: (1) quantify VTE RAM use and variation across physicians and hospitals; (2) assess the relationship between RAM use and chemoprophylaxis use; (3) assess concordance between VTE risk as calculated by the RAM and chemoprophylaxis use; and (4) identify factors associated with discordance between calculated VTE risk and chemoprophylaxis use.

## METHODS

### Sample

We collected electronic health record (EHR) data from medical inpatients ≥18 years of age who were admitted from December 16, 2020, to March 31, 2023 at 10 hospitals within the Cleveland Clinic health system. Patient encounters were included if they used a general admission order set. Encounters where prophylaxis was contraindicated due to therapeutic anticoagulation, high risk of bleeding, or active bleeding, as indicated by the physician in the order set, were excluded. Surgical, ICU, or COVID-positive patients were also excluded. The data were validated manually in an iterative process. For each predictor variable (e.g., smoking status), the investigators reviewed the charts of a random sample of 10 patients who had that variable. When errors were discovered, they were communicated to the data analyst, who amended the code used to extract the variable and a random sample of 10 patients of the new data was reviewed each time. Once we determined that a variable was >90% accurate, we moved on to the next variable. This study was approved by the Cleveland Clinic Institutional Review Board with a waiver of informed consent (22–321).

### Predictor Variables

**Figure 1:**
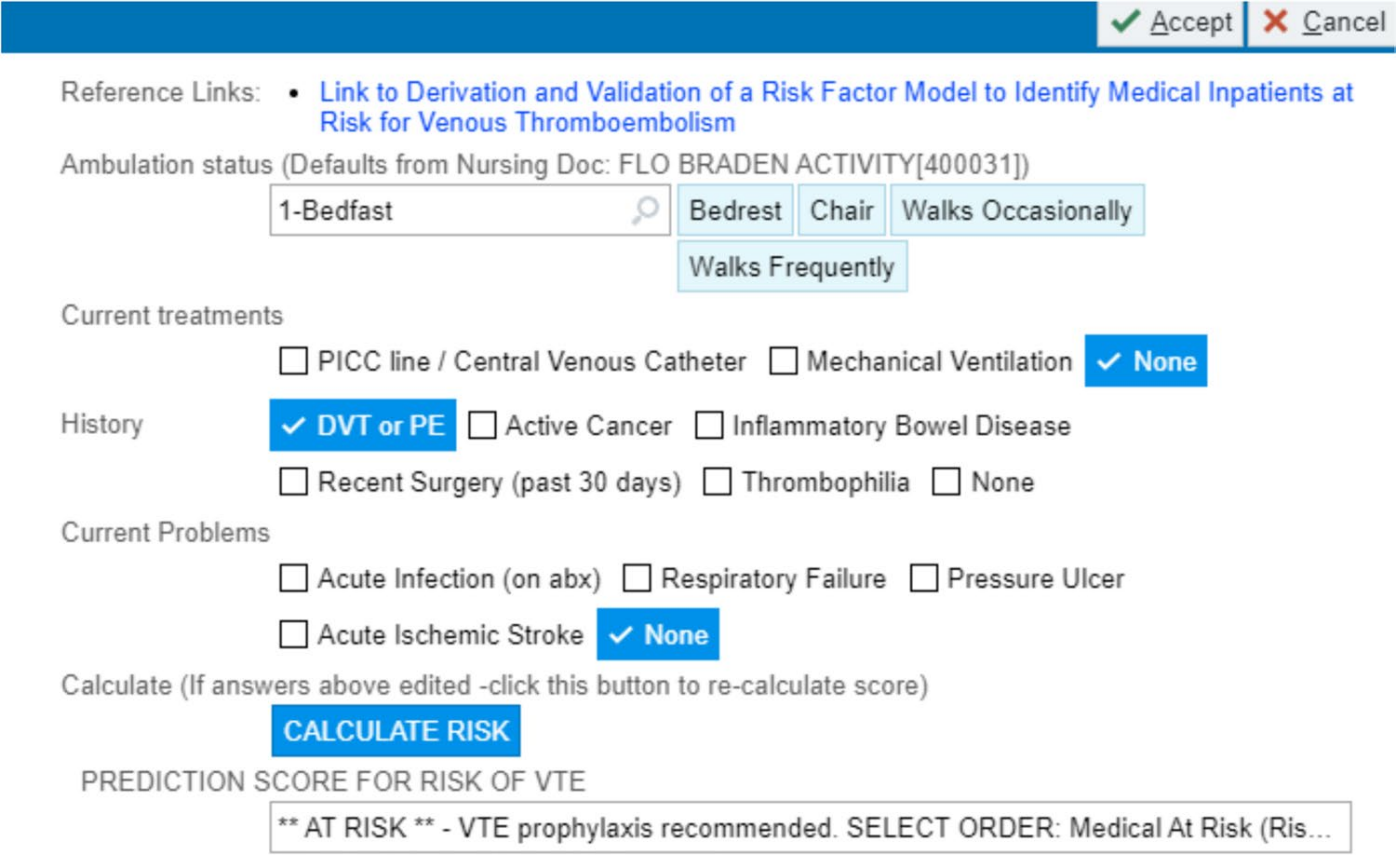
Screen shot of the RAM.

Our primary predictor was a patient’s risk of VTE as determined by the Cleveland Clinic RAM. When physicians enter admission orders using any hospital order set, they cannot pass the VTE module without launching the RAM. They are then prompted to input the risk factors (Fig. 1): activity level, central venous catheter, and mechanical ventilation; history of DVT or PE, active cancer, inflammatory bowel disease, recent surgery, and thrombophilia; and current admission with acute infection, respiratory failure, pressure ulcer, and acute ischemic stroke. The RAM then calculates the risk of developing VTE within 14 days via a logistic regression model. For ease of interpretation, the risk is dichotomized into high and low using a cutoff of 0.75%. The cutoff was determined as the value that maximized the Youden Index (sensitivity plus specificity). The individual risk factors in the RAM were used as potential predictors of discordance between the RAM’s calculated risk (high or low) and prescription of prophylaxis. Discordance was defined as prescription of prophylaxis to a low-risk patient or non-prescription to a high-risk patient. Age, sex, race, smoking status, body mass index (BMI), heart failure, and hospital are not included in the RAM because they do not improve its predictive accuracy, but they were collected for this study because physicians may still consider them, and therefore they may be associated with discordance.

### Outcomes

The primary outcome was chemoprophylaxis ordering, defined as at least one dose of low molecular weight heparin, unfractionated heparin, or fondaparinux ordered within 2 days of admission. Prophylaxis ordering was analyzed at the patient, physician, and hospital level. We also assessed discordance with the recommendation of the RAM, as described above.

### Statistical Analysis

First, the use of the VTE RAM was summarized across hospitals. Next, the percent of patients who had prophylaxis ordered was calculated dichotomously for high-risk and low-risk patients and compared across hospitals and physicians. The percentage of patients for whom the VTE RAM was used was compared to the percentage of patients for whom prophylaxis was ordered using Pearson’s chi-squared test of association.

Second, to understand the relationship between calculated risk and prophylaxis ordering, the percent of patients who had prophylaxis ordered was also calculated for every 0.1% increase in risk of VTE. Then, the physician’s response to the RAM was deemed to be “concordant” or “discordant” depending on the patient’s calculated risk and whether prophylaxis was ordered. Concordance was summarized across calculated VTE risk, physicians, and hospitals. At the physician level, concordance rate was calculated only for physicians with 25 or more encounters during the study period. Concordance rates were also compared across hospitals.

Because predictors of concordance were likely to differ if patients were high-risk versus low-risk, patients were split into two cohorts based on calculated risk. In the stratified cohorts, demographic variables were compared among those who were ordered and not ordered prophylaxis, using Wilcoxon’s rank-sum type or Pearson’s chi-squared test to test for association for continuous and categorical variables, respectively.

Finally, a multivariable logistic regression model was constructed to predict discordance in low-risk patients. Because almost all high-risk patients received prophylaxis, we did not model the predictors of discordance among high-risk patients. All measured characteristics were included in the model.

All analyses were conducted using R version 4.4.0.

## RESULTS

**Figure 2:**
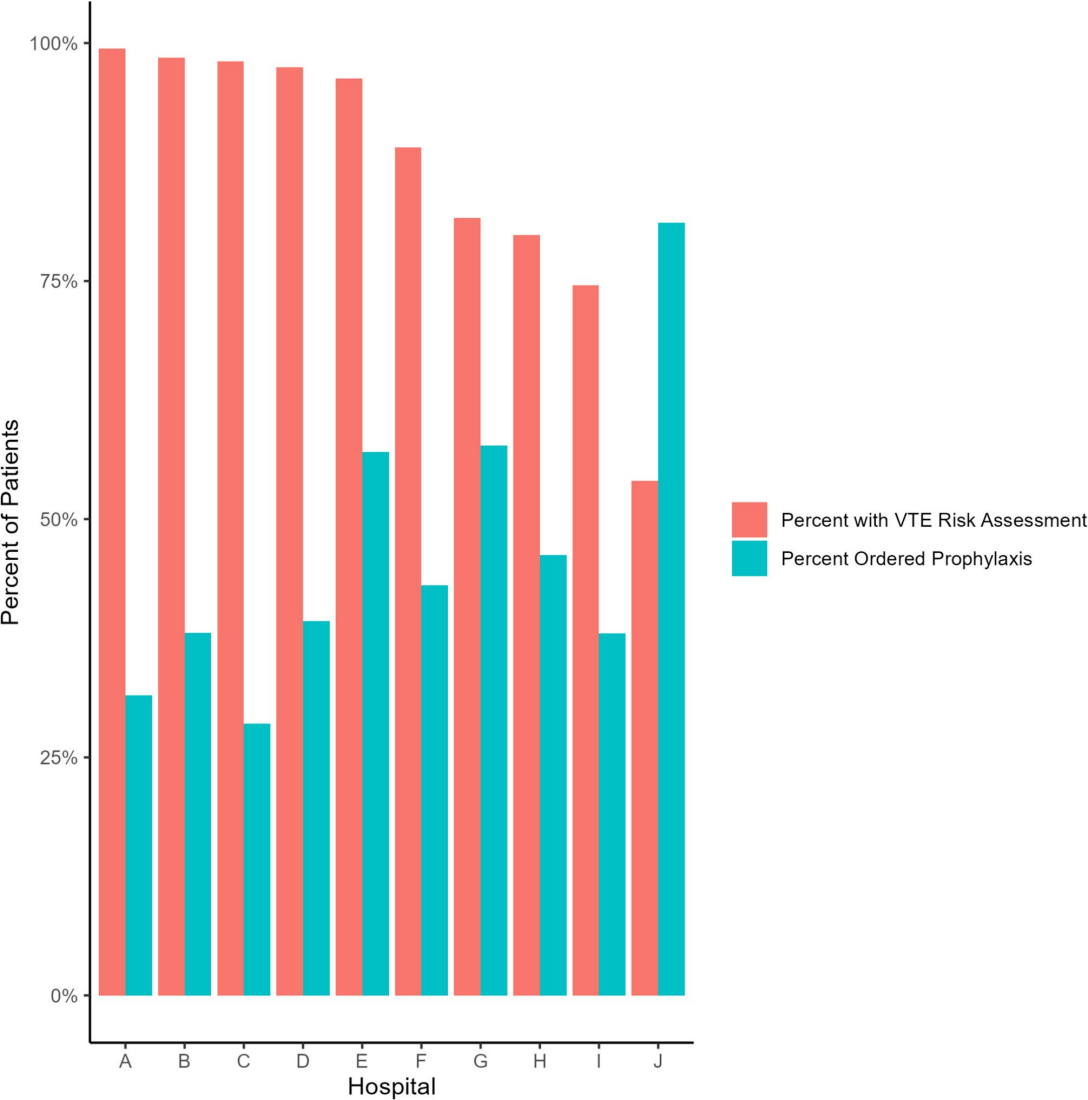
RAM usage and prophylaxis ordering by hospital.

After exclusions (Supplementary Fig. 1), there were 131,441 medical patients admitted from December 16 th, 2020, to March 31, 2023. Despite the VTE RAM being required, only 90% of patients had the RAM’s calculated probability of VTE recorded in the EHR, indicating that some physicians discovered a way to circumvent the “hard stop.” This percentage varied from 54 to 99% across the 10 hospitals (Fig. 2). RAM usage increased over the course of the study period, from 87% in the first half of 2021 to 98% the first quarter of 2023 (Supplementary Fig. 2). Physician RAM use ranged from 0 to 100% for the 862 physicians with 25 or more patient encounters; a majority (72%) of physicians used the RAM for all encounters, and less than 1% never used the RAM.

Within 2 days of admission, 61,910 (47.1%) patients were ordered prophylaxis. Prophylaxis was ordered less frequently when the VTE RAM was used than when it was not (44% vs. 73%, *p* < 0.001). At the hospital level, use of the RAM was inversely related to prophylaxis rate (*r* = −0.76, *p* = 0.01). Of those with a calculated risk score, 15,344 (13%) were high risk, and the remainder were low risk. Prophylaxis was ordered for 96% of high-risk patients versus 37% of low-risk ones. This, too, varied by hospital. The percentage of low-risk patients who received prophylaxis ranged from 21 to 77%, while for high-risk patients the percentage ranged from 87 to 98%.

**Figure 3:**
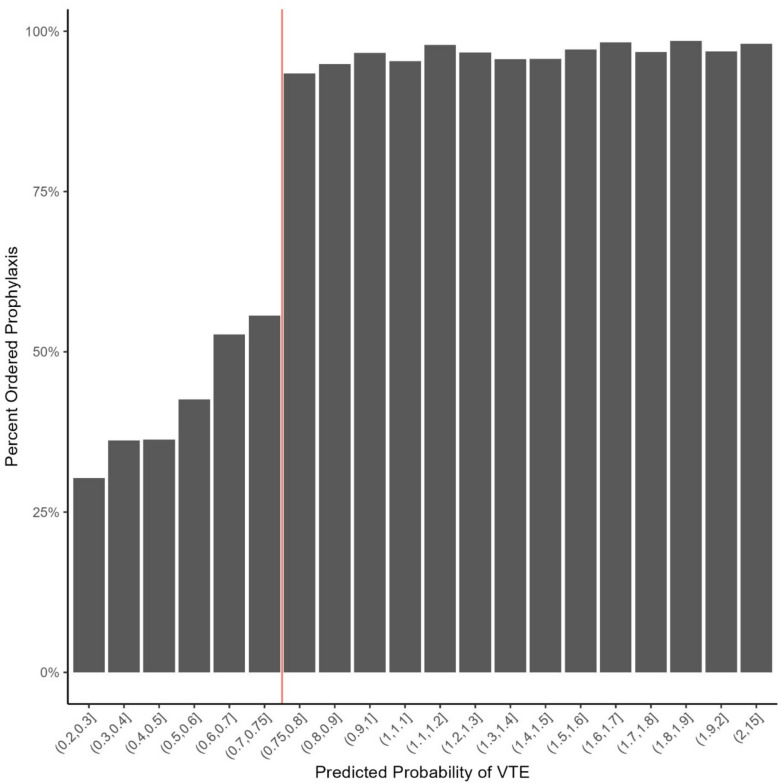
Percent of patients ordered prophylaxis by calculated probability of VTE. The vertical red line indicates the treatment threshold incorporated into the RAM. Patients with calculated probability of VTE >0.75% are categorized as “high risk.”

Figure 3 shows the percent of patients who were ordered prophylaxis as a function of calculated probability of VTE. Above the RAM’s threshold, where prophylaxis was recommended, the percentage of patients who had a prophylaxis order was independent of risk with 95% of patients receiving prophylaxis. Below the threshold, where prophylaxis was not recommended, prophylaxis ordering declined with risk. However, even at the lowest risk interval (0.2–0.3%), 30% of patients had prophylaxis ordered. At the physician level, for physicians with at least 25 low-risk encounters, prophylaxis ordering ranged from 1.75 to 100% (IQR, 32%, 76%), with 2% (*n* = 18) of physicians always ordering prophylaxis. Supplementary Figure 3 shows the relationship between physician RAM use and physician prophylaxis orders. Greater RAM use was associated with lower overall rates of prophylaxis, but there was extreme variability in prophylaxis rates, even among those physicians who used the RAM 100% of the time.

Overall, physician ordering was concordant with the RAM’s recommendation for 68% of patients, 96% of high-risk patients, and 63% of low-risk patients. Concordance with the RAM for physicians with 25+ encounters of low-risk or high-risk patients ranged from 0 to 100% (IQR, 38%, 79%) for low-risk patients and 0 to 100% (IQR, 95%, 100%) for high-risk patients. Mean physician concordance was greater for high-risk patients than for low-risk ones (94% vs. 57%, *p* < 0.001). Across hospitals, too, concordance was greater for high-risk patients (87 to 98%) than for low-risk ones (23 to 79%).

Table 1 describes the patient characteristics and risk factors among those with a calculated probability of VTE, separated by calculated risk. Among low-risk patients, those who were ordered prophylaxis had higher calculated probability of VTE (0.4% vs. 0.37%), and were more likely to be male, older, and less mobile than those not ordered prophylaxis. They were also more likely to have history of DVT, heart or respiratory failure, or active cancer. Being admitted to certain hospitals was also associated with receiving prophylaxis despite being low risk. Among high-risk patients, those who were not ordered prophylaxis (discordant) had lower calculated VTE risk and were more mobile and less likely to have a history of heart or respiratory failure, pressure ulcers, or active cancer.

**Table 1:** Characteristics of High- and Low-Risk Patients by Prophylaxis Status

Characteristic	Low-risk patients, *N* = 102,875			High-risk patients, *N* = 12,798		
	Prophylaxis not ordered *N* = 65,302	Prophylaxis ordered *N* = 37,573	*p*	Prophylaxis not ordered *N* = 609	Prophylaxis ordered *N* = 14,735	*p*
Calculated probability of VTE (%)	0.37 (0.14)	0.40 (0.14)	<0.001	1.11 (0.60)	1.28 (0.81)	<0.001
Age	61 (19)	65 (17)	<0.001	66 (19)	66 (17)	0.4
Gender			<0.001			0.5
Male	28,468 (44%)	17,819 (47%)		273 (45%)	6829 (46%)	
Female	36,816 (56%)	19,751 (53%)		336 (55%)	7904 (54%)	
Other	0 (0%)	0 (0%)		0 (0%)	1 (<0.1%)	
Unknown	3 (<0.1%)	2 (<0.1%)		0 (0%)	1 (<0.1%)	
Nonbinary	15 (<0.1%)	1 (<0.1%)		0 (0%)	0 (0%)	
Race			<0.001			0.1
White	46,006 (70%)	24,672 (66%)		438 (72%)	10,218 (69%)	
Black	13,803 (21%)	10,388 (28%)		128 (21%)	3624 (25%)	
Other	1030 (1.6%)	602 (1.6%)		13 (2.1%)	210 (1.4%)	
Multiracial	3122 (4.8%)	1351 (3.6%)		17 (2.8%)	471 (3.2%)	
Unknown	1341 (2.1%)	560 (1.5%)		13 (2.1%)	212 (1.4%)	
Mobility			<0.001			<0.001
Walks frequently	31,204 (48%)	15,320 (41%)		59 (9.7%)	1231 (8.4%)	
Walks occasionally	28,323 (43%)	17,629 (47%)		230 (38%)	6752 (46%)	
Chair	2280 (3.5%)	1850 (4.9%)		136 (22%)	3142 (21%)	
Bedrest	3495 (5.4%)	2774 (7.4%)		184 (30%)	3610 (24%)	
Smoking status			<0.001			0.9
Not current smoker	52,662 (81%)	31,426 (84%)		518 (85%)	12,494 (85%)	
Current smoker	12,640 (19%)	6147 (16%)		91 (15%)	2241 (15%)	
BMI			<0.001			0.9
Underweight	3339 (5.1%)	2063 (5.5%)		61 (10%)	1153 (7.8%)	
Normal weight	20,310 (31%)	11,249 (30%)		226 (37%)	4758 (32%)	
Overweight	18,258 (28%)	10,532 (28%)		160 (26%)	3712 (25%)	
Obese	23,221 (36%)	13,603 (36%)		161 (26%)	5081 (34%)	
Unknown	174 (0.3%)	126 (0.3%)		1 (0.2%)	31 (0.2%)	
Acute infection	15,660 (24%)	7976 (21%)	<0.001	430 (71%)	10,425 (71%)	>0.9
Acute ischemic stroke	437 (0.7%)	584 (1.6%)	<0.001	4 (0.7%)	135 (0.9%)	0.5
Heart failure	2188 (3.4%)	2179 (5.8%)	<0.001	46 (7.6%)	1825 (12%)	<0.001
Pressure ulcer	151 (0.2%)	118 (0.3%)	0.012	2 (0.3%)	454 (3.1%)	<0.001
Respiratory failure	1588 (2.4%)	1915 (5.1%)	<0.001	106 (17%)	4158 (28%)	<0.001
PICC line	173 (0.3%)	131 (0.3%)	0.017	109 (18%)	2648 (18%)	>0.9
Active cancer	2507 (3.8%)	3342 (8.9%)	<0.001	89 (15%)	3779 (26%)	<0.001
DVT or PE	221 (0.3%)	404 (1.1%)	<0.001	91 (15%)	1802 (12%)	0.046
Inflammatory bowel disease	836 (1.3%)	557 (1.5%)	0.007	12 (2.0%)	530 (3.6%)	0.033
Recent surgery (past 30 days)	692 (1.1%)	402 (1.1%)	0.9	30 (4.9%)	690 (4.7%)	0.8
Thrombophilia	210 (0.3%)	121 (0.3%)	>0.9	17 (2.8%)	305 (2.1%)	0.2

Table 2 shows the adjusted odds ratios of discordance among low-risk patients. Older, male, non-white, and higher BMI patients had significantly higher odds of discordance, but these were weak associations. Presence of most risk factors used in the RAM significantly increased the odds of discordance, in particular history of DVT or PE. Odds of discordance also varied greatly across the hospitals, with one hospital having an odds of discordance that was 7.5 times that of the reference hospital.

**Table 2:** Logistic Model—Predicting Discordance: Low-Risk Patients

Characteristic	OR	95% CI	*p*-value
Age	1.01	1.01, 1.01	<0.001
Gender			<0.001
Female (ref)	—	—	
Male	1.17	1.13, 1.20	
Nonbinary	0.85	0.11, 5.32	
Unknown	0.14	0.01, 0.73	
Race			<0.001
White (ref)	—	—	
Black	1.13	1.09, 1.17	
Other	1.05	0.95, 1.17	
Multiracial	0.91	0.85, 0.97	
Unknown	0.94	0.84, 1.04	
Hospital			<0.001
Hillcrest (ref)	—	—	
Main campus	1.86	1.77, 1.95	
Avon	0.63	0.60, 0.67	
Euclid	0.85	0.79, 0.92	
Fairview	1.01	0.96, 1.06	
Lutheran	0.68	0.63, 0.75	
Marymount	1.05	0.98, 1.12	
Medina	0.81	0.77, 0.86	
South Pointe	7.54	6.90, 8.24	
Weston	2.35	2.23, 2.48	
Mobility			<0.001
Walks frequently (ref)	—	—	
Walks occasionally	1.21	1.18, 1.25	
Chair	1.55	1.45, 1.66	
Bedrest	1.47	1.39, 1.56	
Smoking status			0.004
Not current smoker (ref)	—	—	
Current Smoker	0.95	0.91, 0.98	
BMI			<0.001
Normal weight (ref)	—	—	
Underweight	1.04	0.97, 1.11	
Overweight	1.09	1.06, 1.13	
Obese	1.27	1.22, 1.31	
Heart failure	1.65	1.55, 1.76	<0.001
VTE risk factors included in the RAM			
Acute infection	1.09	1.06, 1.13	<0.001
Ischemic stroke	2.29	2.01, 2.60	<0.001
Mechanical ventilation			
Pressure ulcer	1.24	0.97, 1.60	0.09
Respiratory failure	2.46	2.29, 2.64	<0.001
Central venous catheter	1.6	1.26, 2.02	<0.001
Active cancer	2.34	2.21, 2.47	<0.001
History of DVT or PE	4.62	3.90, 5.48	<0.001
Inflammatory bowel disease	1.64	1.47, 1.84	<0.001
Recent surgery	1.15	1.01, 1.30	0.04
Thrombophilia	1.23	0.97, 1.55	0.09

## DISCUSSION

In this cross-sectional study conducted at 10 hospitals within one health system, we found that physicians used an embedded VTE RAM for the vast majority of patients, but usage varied greatly by individual physician and hospital. When physicians did use the RAM, they were less likely to prescribe prophylaxis, which strongly suggests they were influenced by it. Physicians were more likely to agree with the RAM when it designated a patient as high-risk than low-risk, and patients with certain risk factors were more likely to be prescribed prophylaxis, even if they had a low calculated probability of VTE. Among low-risk patients, those at lowest risk were least likely to receive prophylaxis, but even those with a risk of <0.3% still were prescribed prophylaxis 30% of the time.

Many studies have demonstrated that electronic alerts can improve the use of prophylaxis for high-risk patients.^17^ But physicians generally ignore the alerts. Nendaz et al.^14^, Baroletti et al.^15^ and Kucher et al.^16^ all studied electronic alerts triggered when a high-risk patient was not prescribed prophylaxis. Physicians responded to the alerts only 30 to 38% of the time. In a follow-on study,^18^ in which the electronic alerts were replaced by a page to the attending physician, the human alert resulted in 46% of intervention patients receiving prophylaxis. Our study demonstrates the inclusion of a hard stop can achieve much greater engagement—usage rates in excess of 90% and a prophylaxis rate of 96% for high-risk patients; however, while some physicians routinely accepted the recommendations not to prophylax low-risk patients, others did so selectively. One crucial difference between our approach and those of past alerts is that we presented the physician with risk information before the physician committed to whether the patient was high-risk. Although these approaches have not been directly compared, it makes sense that physicians would be more open to such information before they have made a prophylaxis decision rather than afterward.

As hospitals have successfully increased prophylaxis for high-risk patients, there is growing concern about overtreatment for low-risk ones.^6,7,19^ Reviewing 90,429 medical inpatients at one hospital, Djulbegovic et al. found that 87% of low-risk patients (based on the IMPROVE score) received prophylaxis.^7^ In 2018, Grant et al. examined 44,775 patients across 52 Michigan hospitals and found that overuse of prophylaxis, based on the Padua score, was three times as common as underuse.^19^ Pavon et al., also applying the Padua score, found that almost three-quarters received prophylaxis, with low-risk patients slightly more likely to receive it than high-risk ones.^6^ These rates were similar to what we observed when physicians did not use the RAM—they prescribed prophylaxis to 73% of patients.

Clinical decision support employing a RAM can successfully discourage prophylaxis of low-risk patients. One small study incorporated the Padua model into a templated admission note. Although physicians rarely completed the template, inappropriate prophylaxis for low-risk patients was reduced.^20^ In our study, use of the RAM was associated with reduced prophylaxis among low-risk patients. Overall, 37% received it, although this varied widely across physicians and hospitals. Some physicians prescribed prophylaxis for almost all their patients, but most prescribed it along a gradient, with patients at higher risk more likely to receive it. It is encouraging that physicians did not blindly follow the RAM but added their own judgment. Unfortunately, the gradient was not as steep as one might hope. Many physicians failed to recognize patients at extremely low risk (<0.3%), and consequently many of these received prophylaxis. This may reflect physicians’ misunderstanding of risk, distrust of the RAM, underestimation of the harms associated with prophylaxis, or fears of malpractice litigation, should a patient develop VTE while not on prophylaxis.^21^ The fact that individual risk factors were strongly associated with prophylaxis among low-risk patients suggests that physicians may have trouble distinguishing between high risk as a category (i.e., someone with a risk factor) and as a probability (i.e., greater than some threshold), or they may not have trusted the model when it told them a patient with a well-known risk factor was nevertheless low risk.

More study is needed to discover how to consistently reduce prescribing for patients at very low risk. Meanwhile, hospitals planning on implementing decision support should be aware that physicians may differ widely in the embrace of this technology. In addition, the specific hospital was also a strong predictor of concordance with the RAM’s recommendation. This highlights the influence of local culture on the adoption of new technology.

Others have recognized the importance of making risk assessments mandatory.^22^ Although we included a hard stop for the RAM in all order sets, we were surprised to find that at one hospital 45% of patients were admitted without it. We then discovered that there were at least two ways to circumvent the RAM: de-selecting the RAM in the order section or simply admitting a patient without using an order set. These methods did not explain all the exceptions, however. Additional programming will be required to ensure consistent usage.

Our study has several limitations. First, the data were extracted from the EHR and may contain some errors. We performed validation to assure that each variable was greater than 90% accurate, and therefore such errors should be small. Second, our data were from a single hospital system with a shared EHR, so our results may not be generalizable to other systems. However, we did include 10 hospitals which varied substantially in size, patient acuity, patient demographics, geographical location, and physician demographics. The variation which we observed is likely representative of the degree of variation which may be observed in other hospitals. Our study was cross sectional, so we cannot prove causality. However, the reduction in prophylaxis when the RAM was used (44% vs. 73%) was remarkably similar to that seen in the per protocol analysis of the randomized trial (47% vs. 75%).^13^ Lastly, we did not assess outcomes, so we do not know whether those physicians who prescribed more prophylaxis had better outcomes. However, based on previous studies in our hospitals (both the RCT and a longitudinal study), this does not appear to be the case.

Overall, use of a validated RAM was associated with lower prophylaxis use, particularly among low-risk patients, but there was substantial variation in use among physicians and hospitals. Even when they used the RAM, physicians varied in their use of prophylaxis, especially for patients designated as low risk by the RAM. Many low-risk patients, particularly those with certain risk factors for VTE, were still prescribed prophylaxis. More study is needed to understand the sources of this variation in order to reduce overuse of prophylaxis for low-risk patients.

## Supplementary Information

Below is the link to the electronic supplementary material.

Supplementary file1 (DOCX 337 KB)

## Data Availability

Data are not available to share with investigators outside of Cleveland Clinic.
