# Supplementary material for: Use of a Risk Assessment Model for Venous Thromboembolism Is Associated with Decreased Prophylaxis
Source: J Gen Intern Med. 2025 May 8;40(15):3649–57. doi: 10.1007/s11606-025-09592-6 (PMC12612495; doi:10.1007/s11606-025-09592-6)

Use of a risk assessment model for venous thromboembolism is associated with decreased prophylaxis

Tarini Gunaratne, MD^1^, Rebecca Schulte, MPH^2^, Stephanie Moss, MD^3^, Oleg Lisheba, MS,^4^ **Michael B. Rothberg**, MD, MPH^5^

Supplementary Materials

Supplementary Figure 1: Enrollment Flow Diagram


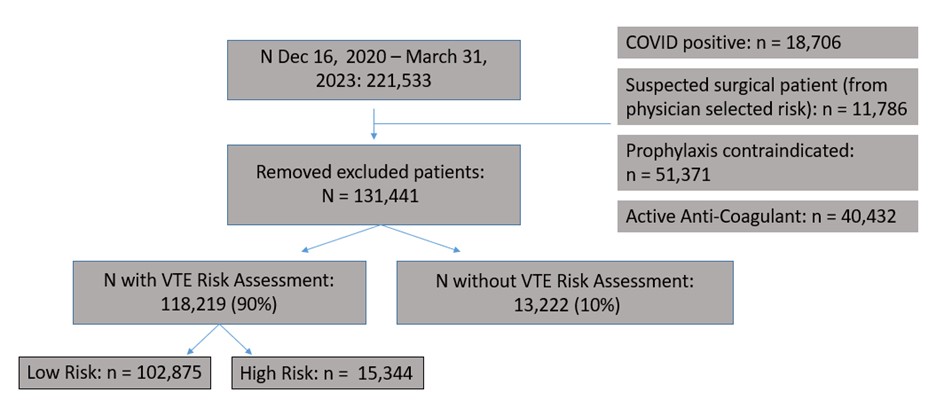


Supplementary Figure 2 – RAM usage over time at each of the study hospitals


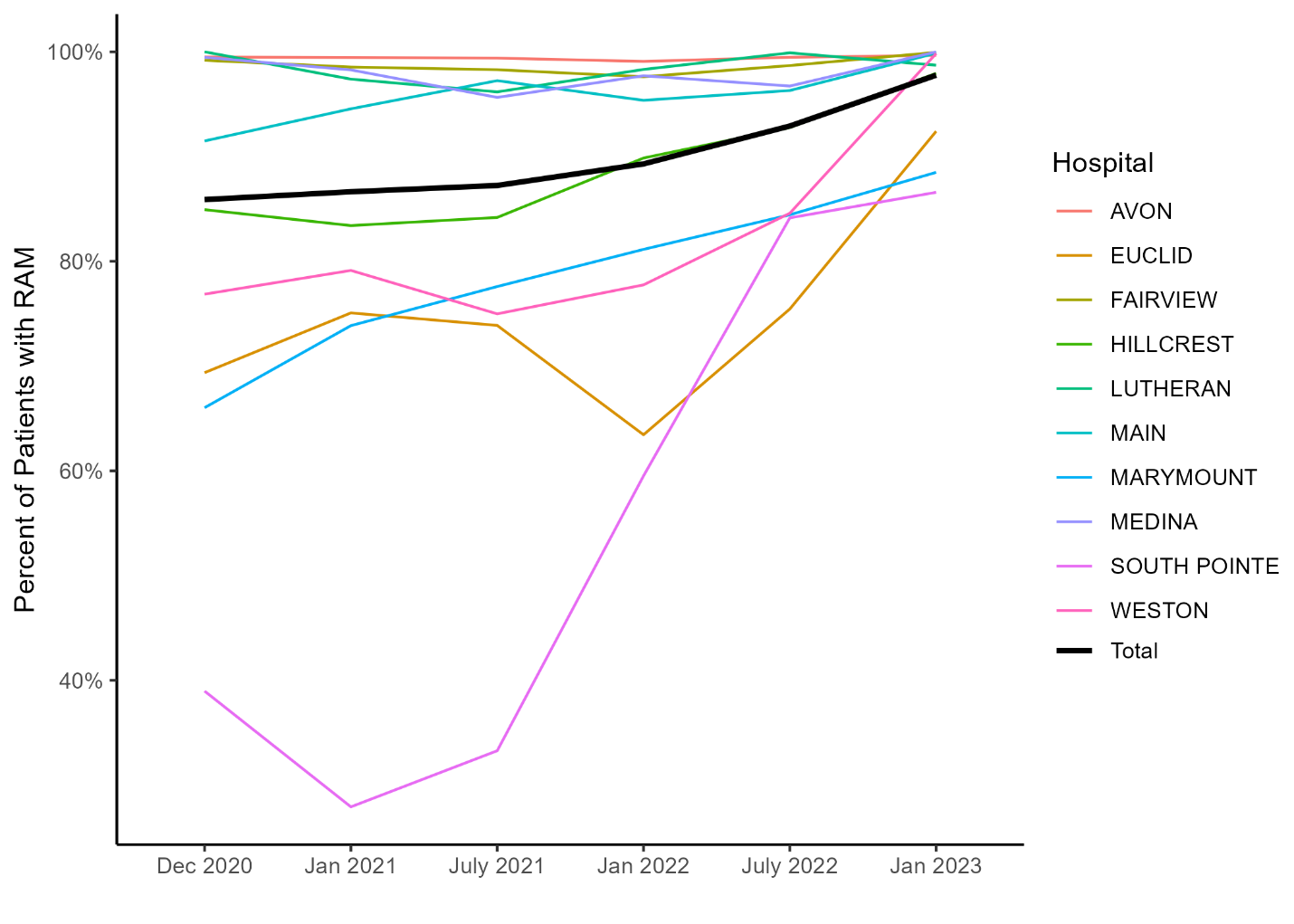


Supplementary Figure 3 – Physician RAM use vs physician prophylaxis ordering
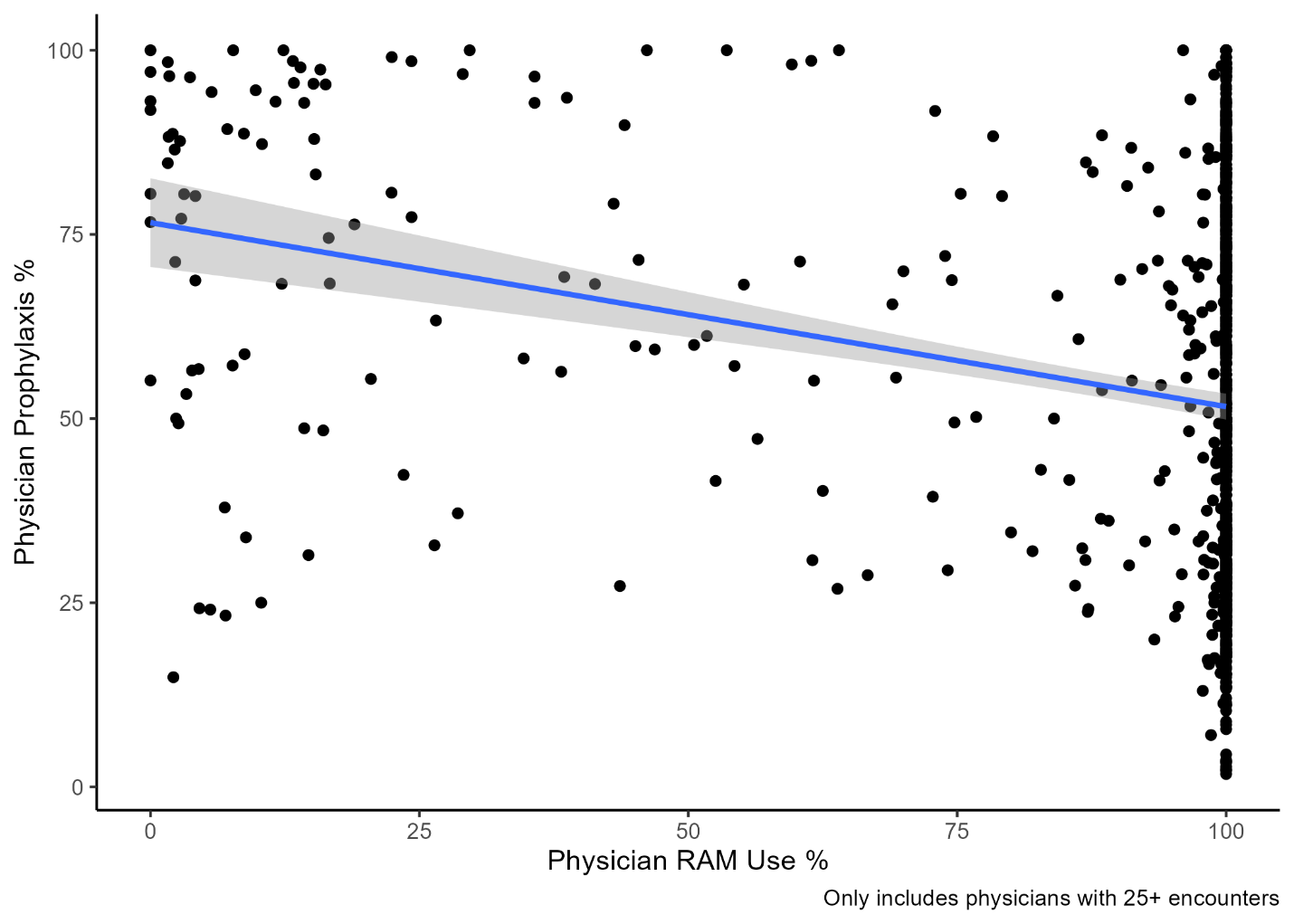

Supplement: Supplementary file 1 — Supplementary file1 (DOCX 337 KB) [file 11606_2025_9592_MOESM1_ESM.docx]
